# Early prevention by L-Arginine attenuates coronary atherosclerosis in a model of hypercholesterolemic animals; no positive results for treatment

**DOI:** 10.1186/1743-7075-6-13

**Published:** 2009-03-24

**Authors:** Shaghayegh Haghjooy Javanmard, Mehdi Nematbakhsh, Mohammad Hosein Sanei

**Affiliations:** 1 Applied Physiology Research Center and Department of Physiology, School of Medicine, Isfahan University of Medical Sciences, Isfahan, Iran; 2 Department of Pathology, School of Medicine, Isfahan University of Medical Sciences, Isfahan, Iran

## Abstract

**Background:**

Endothelial dysfunction (ED) is an independent predictor of cardiovascular events. ED is also a reversible disorder, and nitric oxide donors like L-arginine may promote this process. Despite the positive results from several studies, there are some studies that have shown that L-arginine administration did not improve endothelium-dependent dilation or the inflammatory state of patients. In this study the early and the late effects of L-arginine on coronary fatty streak formation and ED biomarkers were considered in hypercholesterolemic rabbits.

**Methods:**

36 white male rabbits randomly assigned in 3 groups. Rabbits were fed 1% high-cholesterol diet (LP group, n = 15), or high-cholesterol diet with oral L-arginine (3% in drinking water) (EP group, n = 15) or standard diet (control group, n = 6) for 4 weeks (phase I). Afterward, all animals were fed normal diet for 4 weeks (phase II). In the second phase, L-arginine was discontinued for EP group and was begun for LP group. The plasma levels of lipids, von Willebrand factor (vWF), and nitrite were compared before and after 4 and 8 weeks of experiment. Coronary fatty streak formation was measure after 4 and 8 weeks of experiment.

**Results:**

The plasma levels of lipids were increased significantly in both groups of LP and EP after phase I. The hypercholesterolemia induced significant increased vWF release in LP group. The L-arginine supplementation led to significant plasma nitrite increment in EP group. The vWF in LP group was higher than other groups (p < 0.05). By the end of phase II, despite of start of L-arginine supplementation for LP group and L-arginine discontinuation in EP group, there were significantly more fatty streaks lesions in LP group coronary arteries than EP group. Furthermore, L-arginine supplementation did not result in significant nitrite increment in LP group.

**Conclusion:**

Early prevention by L-arginine may be helpful to prevent the ED, but our study did not suggest the treatment. It seems reasonable to consider ED-aside from control the cardiovascular risk factors in primary prevention of atherosclerosis and its clinical outcomes before development of irreversible vascular damage.

## Background

Atherosclerosis is the single most important cause of cardiovascular disease (CVD) – a predominant health problem worldwide[1]. Clinical manifestations of atherosclerosis include myocardial infarction, heart failure, stroke, and peripheral artery disease, result in irreversible organ damage[2]. Early atherosclerosis lesions or fatty streaks become increasingly prevalent among children and young adults in industrialised countries[3,4]. Fatty streaks lesion formation may begin even before birth as intimal thickening can be observed in fetal coronary arteries[5]. Although these lesions may be vanishing, some of these lesions progress to advanced stages of atherosclerosis. As atherosclerosis has a long asymptomatic phase and the first manifestation of disease may be sudden cardiac death, it is imperative to find effective strategies to prevent it[4,6]. Current guidelines for the prevention of atherosclerotic diseases focus on treatment of established cardiovascular risk factors to attenuate the subsequent endothelial cell dysfunction and damage[6]. Endothelial dysfunction (ED) is an early event in atherosclerosis and has a pivotal role in the atherogenesis process[7].

ED is an independent predictor of cardiovascular events, therefore it seems reasonable to be mentioned, aside from risk factor modification in primary prevention[6,7]. Furthermore ED is a reversible disorder and search for the proper time before development of irreversible vascular injury is extremely important [4].

ED is characterized by reduced bioavailability of nitric oxide (NO) [8-11]. NO is a potent anti atherosclerotic molecule and every intervention that enhances NO bioavailability might be a promising strategy for the prevention and treatment of atherosclerosis [10-13]. One straightforward approach to increase NO bioavailability is providing additional substrate for nitric oxide synthase (NOS). L-Arginine is the substrate of endothelial NOS (eNOS) and the main precursor of NO in the vascular endothelium. Data from numerous studies imply that L-arginine supplementation restores endothelial function in several disease states associated with ED such as hypercholesterolemia [14-18]. Despite the positive results from several studies, there are some studies that have shown that L-arginine administration did not improve endothelium-dependent dilation or the inflammatory state of patients [18]. L-arginine could even be harmful to vascular health; for example L-arginine supplementation during the post-myocardial-infarction period was associated with higher post-infarction mortality than placebo [19]. So, it is still unclear whether administration of L-arginine has any beneficial effect on clinical outcome [18].

The present study is aimed to investigate the preventive role of L arginine in two approaches: 1) L arginine supplementation concurrently with cholesterol enriched diet, and 2) L arginine supplementation four weeks later than cholesterol enriched diet initiation and fatty streaks formation in hypercholesterolemic rabbit's aortas.

## Methods

### Animals and Experimental design

This study was reviewed and approved by the Ethics Committee of Isfahan University of Medical Sciences. 36 white male rabbits were obtained from the Razi Institute of Iran. After 1-week acclimation period, and an overnight fasting blood samples were taken as pre experiment sampling. Collected blood samples were centrifuged (10,000 _ *g*), and the resulting plasma was stored at -70°C until measurements. The animals were then randomly assigned in 3 groups. The rabbits were fed rabbit chow supplemented with 1% cholesterol (Hypercholesterolemic diet, Late prevention group; LP, n = 15) or high-cholesterol diet with oral L-arginine (3% in drinking water; Early prevention group; EP, n = 15) or standard diet (control group, n = 6) for 4 weeks. The cholesterol-enriched diet was prepared daily on site by dissolving cholesterol in the olive oil and thoroughly coating the pellets of rabbit chow with this mixture [20]. The olive oil was also added to the pellets of rabbit chow of control group. Each animal in each group had daily access to 120 g of pellets. Water was available ad libitum. The cholesterol and L-arginine were purchased from Scharlau (Barcelona, Spain) and Ajinomoto Co (Japan) respectively. The body weight determined before experiment and at 4^th ^and 8^th ^weeks.

By the end of 4 weeks (phase I), the blood samples were taken and stored again. Ten animals from each of EP and LP groups and three animals from control group randomly were selected and euthanized by an overdose of sodium pentobarbital and exsanguinated. The animal's hearts were harvested for pathological investigation. From this point, hypercholesterolemic diet was break off and all remainder animals were on normal diet, L-arginine supplementation for EP group was stopped and oral L-arginine supplementation (3% in drinking water) for LP group was began. The experiment was continued for another 4 weeks (phase II). At the end of the phase II, the blood sampling and storage were repeated. The rest of the animals were scarified and the hearts were harvested for pathological investigation.

### Lipid and lipoprotein measurements

Total cholesterol, triglyceride, high density lipoprotein (HDL) and low density lipoprotein (LDL) cholesterol levels were measured by standard enzymatic kit (Pars Azmoon Co, Iran)

### Plasma vWF and nitrite measurement

Von Willebrand factor (vWF), is a well-established in vivo marker of endothelial cell activation and injury [21]. Plasma vWF was measured by sandwich enzyme-linked immuno-sorbent assay (ELISA) using paired capture and detecting anti-vWF. antibodies (Cedarlane Co, Canada) as previously described [21].

The plasma level of nitrite (stable NO metabolite) was measured using a colorimetric assay kit (R&D Systems, Minneapolis, USA) that involves the Griess reaction as previously described [22].

### Coronary fatty streaks or CSN determination

The hearts were fixed with 10% neutral buffered formalin solution and embedded in paraffin. The paraffin blocks were sectioned serially and serial sections prepared at 1000 μm intervals were stained with haematoxylin and eosin. Using an Olympus microscope equipped for projection, the sampled sections of the coronary arteries were projected onto a table top (at 100× magnification). An orthogonal grid (2 cm × 2 cm) was superimposed over the projected image, and the number of grid points overlying the intima, and lumen for the coronary artery were counted by two independent experts blinded to the experiment groups. The luminal area and the area covered by intimal lesions were delineated. Coronary cross sectional narrowing (CSN) was calculated using the equation: CSN (%) = 100× the surface area of lesions/the luminal area. The CSN shows the degree of fatty streaks formation and atherosclerosis progression[23,24].

### Statistical analysis

The data are reported as the mean ± SEM. A statistical software package, SPSS (version 13), was used to perform statistical analysis. The data were tested for normality and homogeneity of variance. ANOVA repeated measures were used to assess within group changes and One-way ANOVA followed by Bonferroni's *t*-test within groups were used to assess the significance of any change between groups. Non-parametric methods (Wilcoxon rank-sum test and Kruskal-Wallis test) were used for the comparison of CSN, within and between groups. Statistical significance was accepted at *p *< 0.05.

## Results

### The effect of L-arginine on plasma levels of lipids and lipoproteins

The hyperlipidemia induced by dietary cholesterol in rabbits is owing to overproduction of lipoproteins and impaired plasma clearance secondary to the down-regulation of LDL receptor expression in rabbit's liver [25]. The effect of 4 weeks high cholesterol diet on cholesterol and LDL levels have been shown in table 1. Initial values for lipids and lipoproteins were similar in all groups. The cholesterol-rich diet induced a significant increase of total cholesterol, LDL and HDL-cholesterol, and triglyceride (TG) in both EP and LP groups (p < 0.05) through the first phase of study. By the end of phase II, plasma cholesterol, LDL and HDL reduced significantly but did not return to baseline values in both EP and LP groups (Table 1). The plasma TG level reduced significantly and there was no difference between pre experiment and ending values (Table 1). The lipid and lipoprotein levels in the control group remained statistically unchanged through the study. By the end of phase I, the animals of the EP and the LP group had similar levels of cholesterol, LDL, HDL and TG as well as by the end of phase II (p > 0.05); while all of the lipids and lipoproteins in these 2 groups were significantly higher than control group (p < 0.05) (Table 1). These results showed that L arginine had no lipid lowering effects in our study.

**Table 1:** The plasma levels of lipids, lipoproteins, nitrite, vWF and rabbit's body weights in three groups of the study in three sampling sessions.

**Parameter/group**		**EP**	**LP**	**Control**
**Cholesterol** **(mg/dl)**	A	108.56 ± 20.1	94.13 ± 15.4	118.65 ± 12.1
	
	I	1918.93 ± 245.4*	2291.68 ± 227.8*	138.38 ± 11.8 ‡
	
	II	1275.00 ± 169.5* , †	1433.40 ± 161.3*, †	122.7 ± 10.5 ‡

**Triglyceride (mg/dl)**	A	122.61 ± 2.3	121.42 ± 3.9	119.31 ± 2.7
	
	I	228.12 ± 26.7*	232.4 ± 25.6*	121.89 ± 4.3‡
	
	II	147.0 ± 17.2†	141.02 ± 15.6†	117.90 ± 3.2‡

**LDL** **(mg/dl)**	A	28.35 ± 9.4	44.01 ± 10.8	33.5 ± 8.9
	
	I	1079.66 ± 118.2*	1418.99 ± 189.6*	42.76 ± 10.7 ‡
	
	II	623.18 ± 126.5*, †	635.09 ± 113.7*, †	38.6 ± 11.4 ‡

**HDL** **(mg/dl)**	A	15 ± 2.4	15.5 ± 1.3	16.4 ± 1.7
	
	I	109.6 ± 20.1*	96.1 ± 11.6*	18.3 ± 1.4‡
	
	II	60.6 ± 11.2*†	74.0 ± 9.4*†	20.0 ± 3.2‡

**Nitrite** **(μM/l)**	A	11.40 ± 1.0	11.11 ± .8	9.86 ± .8
	
	I	14.7 ± .5*, ‡	12.2 ± .7	9.86 ± .8
	
	II	16.57 ± 1.2*, ‡	14.58 ± 2	8.83 ± .2

**vWF** **(IU/dl)**	A	0.55 ± .06	0.42 ± .09	0.32 ± .04
	
	I	0.49 ± .04	0.99 ± .1 *, ‡	0.35 ± .06
	
	II	.08 ± .006*, †	.07 ± .006*, †	0.23 ± .04 ‡

**Body weight** **(kg)**	A	2.15 ± 0.1	2.13 ± 0.2	1.87 ± 0.3
	
	I	2.37 ± 0.2*	2.38 ± 0.1*	1.92 ± 0.2‡
	
	II	2.51 ± 0.3†	2.45 ± 0.2†	2.0 ± 0.2‡

A: pre-test sampling, I: after 4 weeks (phase I) and II: after 8 weeks (phase II).

significant difference * from (A), † from (I); and ‡ from other groups (p < 0.05).

### The effect of L-arginine on body weight

As shown in Table 1, in the beginning there were no significant body weight differences between three groups of the study. Both cholesterol-fed groups significantly gained weight throughout the study; while there was no significant weight gain in the control group. There was no significant weight gain difference between EP and LP groups through the experiment. Thus, L-arginine did not affect weight gain.

### The effect of L-arginine on plasma level of nitrite

During the first phase, the plasma level of nitrite significantly increased in EP group (p < 0.05), and it was also significantly higher than other groups (p < 0.05) (table 1). During the phase II, despite of the start of L-arginine supplementation for LP group, there was no significant change in nitrite concentration. The plasma nitrite concentration in EP and control groups also remained statistically unchanged in this phase. Finally in phase II, there was no significant difference in plasma level of nitrite, between EP and LP groups; while both groups had significantly higher level of nitrite than control group (table 1).

### The effect of L-arginine on plasma level of vWF

The plasma level of vWF significantly increased during the first phase of experiment in LP group, and it was statistically different from others groups (p < 0.05) (table 1). By the end of phase II, the plasma level of vWF significantly decreased in EP and LP groups while the plasma level of vWF remained unchanged in control groups (p < 0.05) (table 1).

### The effect of L-arginine on CSN; fatty streaks

**Figure 1:**
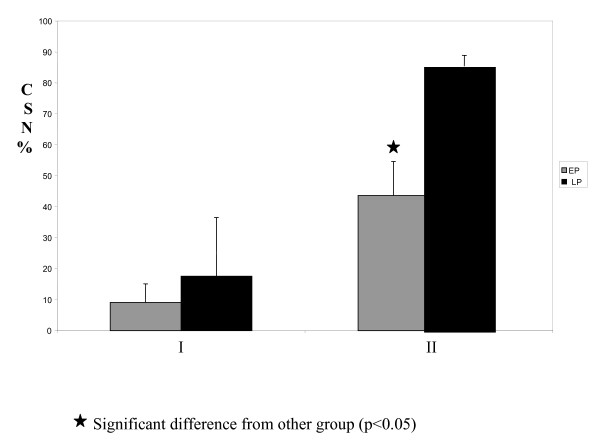
**Comparison of coronary crosses sectional narrowing (CSN) between EP and LP groups; Fatty streaks were seen in both groups of rabbit's coronary arteries by the end of phase I**. In this phase, there was no significant difference in the CSN between the groups; however, by the end of phase II the CSN of the EP group was significantly lower than LP group. I: after 4 weeks (phase I) and II: after 8 weeks (phase II). LP: Late prevention, EP: Early prevention.

Figure 1 shows measurements of the extent of atherosclerotic lesions of the coronary arteries. Early atheromatous lesions (fatty streaks) were seen in rabbit's coronary arteries by the end of phase I. In this phase, there was no significant difference in the CSN between the groups; however, by the end of phase II the CSN of the EP group was significantly lower than LP group. There was no fatty streak lesion in control animals' coronary arteries during the study.

## Discussion

The aims of this study were to investigate the effect of L-arginine as a NO donor on the atherosclerosis prevention and search for the appropriate time for its beginning in a rabbit model of hypercholesterolemia. We used the dietary reversal model of hypercholesterolemia because in a realistic clinical setting the first-step therapy in hypercholesterolemic patients is the lowering of cholesterol; so the LP group is the equivalent of hypercholesterolemic patients and the EP group is the equivalent of patients at risk of developing hypercholesterolemia and atherosclerosis.

The results showed that hypercholesterolemia induced significant endothelial injury characterized by increased vWF release within 4 weeks after initiation of a 1% cholesterol diet in LP group. As we assumed L-arginine supplementation augmented NO production in EP group in the first phase of the study. In spite of the presence of hypercholesterolemia, L-arginine preserved endothelium from adverse effects of risk factor as substantiated by significantly lower vWF in this group. This finding is in line of previous studies in which NO restrained ED and injury [9,11,13]. Several other studies that investigated L-arginine beneficial effects on endothelial function, particularly in hypercholesterolemic animals, have supplemented L-arginine during induction of hypercholesterolemia [17,26-28]. These studies are similar to EP group in our study and attained similar results. These experiments showed that ingestion of L-arginine, as a boosting supplement for NO production can reverse the state of oxidative stress and progression of atherosclerosis. Furthermore, as we have shown in our recently published paper, the positive results of L-arginine on endothelial function may be the consequence of enhanced expression of eNOS in vessel wall [29].

By the end of phase II, significant fatty streak lesions were formed in coronary arteries of LP groups. Despite of the start of L-arginine supplementation for LP group and L-arginine discontinuation in EP group, there were significantly more fatty streaks lesions in LP group coronary arteries than EP group. This finding is interesting in light of other findings that L-arginine supplementation did not result in significant NO increment in LP group. This is in agreement with other studies that have shown that L-arginine did not improve endothelial function in patients with coronary atherosclerosis or myocardial infarction [30-32]. These findings suggest that L-arginine is effective before exhaustive ED and vascular endothelium damage. It seems that L-arginine supplementation is useful in vascular bed with healthy and functional eNOS. It might even be harmful if supplemented in situations with eNOS uncoupling[18].

It is likely that we need to start effectual prevention much earlier than previously assumed. Consistent with this view, finding from paediatric studies complemented by necropsy data demonstrate clearly that children and young adults are also liable to the effects of cardiovascular risk factors and show early signs of atherogenesis[3,5]. Furthermore, it has been shown that maternal hypercholesterolemia is associated with greatly enhanced fatty streaks formation in fetal arteries, both in human and animal models [33-35]. These observations may provide rational for start prevention in childhood.

## Conclusion

As the results of our study showed, L-arginine supplementation after induction of hypercholesterolemia may lead to more coronary fatty streaks formation while L-arginine was effective before exhaustive ED and vascular endothelium damage. Early prevention by L-arginine may be helpful to prevent ED, but our study did not suggest the treatment.

In addition, given the important role of ED for the development of atherosclerosis, it seems reasonable to consider ED-aside from control of the cardiovascular risk factors- as a primary therapeutic target in the prevention of atherosclerotic disease. The observation that interventions such as L-arginine supplementation improve endothelial function without altering the risk factor profile supports the concept that the endothelium serves as a central transducer by which risk factors can mediate progression to atherosclerosis, and its status may reflect the propensity to develop atherosclerotic disease.

This study has several limitations. First, the rabbit model in this study is a rapidly progressive model and we must be cautious when we apply short-time outcome in animal model to human slowly progressive disease, such as atherosclerosis. A second potential limitation of our study is the relatively small sample size. A third potential limitation of the study is using only one dose of oral L arginine. Furthermore, as the present study was designed to assess the effects of L-arginine for 4 weeks, the long-term effects of L-arginine need to be studied.

Further studies are needed to retrieve the right timing to initiate primary prevention of atherosclerosis and its clinical outcomes before development of irreversible vascular damage.

## Competing interests

The authors declare that they have no competing interests.

## Authors' contributions

SHJ had substantial contributions to conception and design, biochemical measurements and immunoassay, analysis and interpretation of data, and writing the manuscript. NM had substantial contributions to conception and design, analysis and interpretation of data. He has also been involved in drafting the manuscript. MHS carried out the pathological investigation. All authors read and approved the final manuscript.
